# Cell-scale atmospheric moisture flows dataset reconciled with ERA5 reanalysis

**DOI:** 10.1038/s41597-025-04964-3

**Published:** 2025-04-15

**Authors:** Elena De Petrillo, Luca Monaco, Marta Tuninetti, Arie Staal, Francesco Laio

**Affiliations:** 1 Department of Environment, Land and Infrastructure Engineering, Politecnico di Torino, Turin, Italy; 2 Copernicus Institute of Sustainable Development, Utrecht University, Utrecht, Netherlands

**Keywords:** Hydrology, Environmental sciences, Scientific data

## Abstract

Water vapour flows in the atmosphere are fundamental to the hydrological cycle, linking evaporation sources to precipitation sinks. Recent atmospheric tracking models have provided valuable insights, allowing one to trace the sources of precipitation and determine where evaporated water from specific regions will eventually precipitate. Despite improvements in model accuracy, there remain significant discrepancies between reconstructed and observed evaporation and precipitation data from reanalysis. To address these discrepancies and enhance the reliability of tracking models’ estimates, we propose a procedure based on Iterative Proportional Fitting (IPF). Using this approach, we reconcile atmospheric moisture flows reconstructed by the Lagrangian model UTrack with ERA5 reanalysis data. This ensures that the traced atmospheric water matches the total evaporation and the precipitation annually. The reconciled bilateral connections provide a new dataset (RECON) centred on the period 2008-2017 that facilitates the exploration of atmospheric vapour flows between evaporation and precipitation basins at the global scale with a spatial resolution of 0.5°. Further, the proposed framework applies to any cell-scale dataset of atmospheric moisture tracking.

## Background & Summary

A key part of the global hydrological cycle is comprised of the moisture flows through the atmosphere, which connect locations where the moisture evaporates with the locations where it subsequently precipitates. Continental moisture recycling is so important for global precipitation patterns that roughly half of all terrestrial precipitation has come from evapotranspiration from land, the other half being from the ocean^1–3^. Moisture recycling connects land- and atmospheric conditions up to thousands of kilometres away. For example, land-use changes that affect evapotranspiration flows, such as deforestation, can affect precipitation regimes, the severity of droughts and hydrological flows in downwind regions^4–9^. Although these connections vary in time, they do occur in quite consistent patterns^1^. Therefore, reconstructing evapotranspiration-to-precipitation connections from the recent past allows us to better understand the role of surface processes in the global hydrological cycle. With this knowledge, we are able to assess the effects that land cover changes may have on precipitation at continental scales.

Because of their relevance to diverse fields, a broad range of researchers has developed interest in the tracking techniques to develop these vapour flows reconstructions. The so-called atmospheric moisture tracking models typically use atmospheric reanalysis data to simulate the atmospheric branch of the hydrological cycle. However, despite the large interest, it is often not feasible for researchers to perform these simulations. As with all methods, becoming familiar with them requires significant time investment, but an additional constraint on widespread use of atmospheric moisture tracking is its heavy data demand. This data demand has increased considerably with the largest generation of atmospheric reanalysis data, ERA5^10^, which allows for obtaining detailed global moisture flows. The highest-resolution global dataset of atmospheric moisture connections between evapotranspiration and precipitation has been generated using the UTrack model^3^. The UTrack model provides a comprehensive database of tracked atmospheric moisture flows, mapping the bilateral connections between water sources (evaporation) and sinks (precipitation)^3^. This database utilizes a Lagrangian (trajectory-based) approach that tracks moisture flows across the globe, including oceans, with output at 0.25° spatial resolution. It includes monthly multi-annual means of atmospheric moisture flows from 2008 to 2017, capturing detailed pathways of moisture transport at a 0.5° spatial resolution globally. These data are vital for understanding the distribution and movement of atmospheric water, serving as a critical resource for researchers studying hydrological cycles and climate dynamics. Recent literature has demonstrated the globality of the water cycle where hydrological flows interact across different scales where globalising stimuli (e.g., climate change or food demand) have repercussions on local water management. This understanding implies the need for reliable confidence in estimating freshwater teleconnections, making it crucial to frame atmospheric moisture flows within the global hydrological cycle and to make it internally consistent.

Trajectory-based moisture tracking research has a long-standing history, with models steadily advancing as cutting-edge data becomes increasingly available^1,3,11–13^. This is the case with the UTrack Lagrangian model^3,14^, which takes advantage of state-of-art climate reanalysis data and, by testing several combinations of model assumptions, optimally generates highly detailed evaporation footprints while avoiding unnecessary complexity. In just several years, the UTrack database has already been widely used. For example, it has been instrumental to quantify effects of global forest restoration on water availabilities^15,16^, to determine moisture flow dependencies between nations^17,18^, to assess the role of forests in global precipitation variabilities^9^, and on agriculture in Africa^19^, as well as to analyse the global hydrological cycle as a network^20^. Despite these cutting-edge model advancements and the wide applications already achieved, less attention has been given to ensuring the consistency of tracked moisture volumes with reanalysis data of precipitation and evaporation simultaneously to ensure the closure of the annual hydrological cycle. Model error and assumptions, as well as possible discrepancies in ERA5 data, may lead to inconsistencies that could impede internally consistent descriptions of the global hydrological cycle.

Indeed, uncertainty related to a set of modeling assumptions and data resolution still poses an issue for the moisture tracking community. All studies that use offline moisture-tracking models make choices regarding vertical mixing of the moisture at the start of the tracking and during its path through the atmosphere, integration time step, interpolation, and resolution of the forcing data set. Further, in each moisture recycling study, assumptions are chosen such that a suitable trade-off is achieved between accuracy of the representation of the downwind evaporation shed (the distribution of precipitation resulting from evaporation from a point or area), amount of data needed, and simulation time^21^. For the UTrack tracking model,^3^ explicitly show model-dependent uncertainties related to (i) the number of parcels in which divide a column of evaporation (ii) release height of moisture (iii) the vertical mixing (iv) the time-step integration (v) horizontal and vertical scale degradation of forcing data and (vi) interpolation. In UTrack, moisture parcel trajectories follow three-dimensional trajectories, meaning that if vertical mixing occurs according to the ERA5 data, it is captured by the parcel trajectories. However, to account for known underestimations of vertical fluxes of atmospheric vapour, a random vertical mixing term was added by Tuinenburg, Staal (2020)^3^: on average every 24 hours, a moisture parcel may be reassigned a new vertical position, the probability of which depends on the atmospheric moisture profile at the current location of the parcel.

**Fig. 1:**
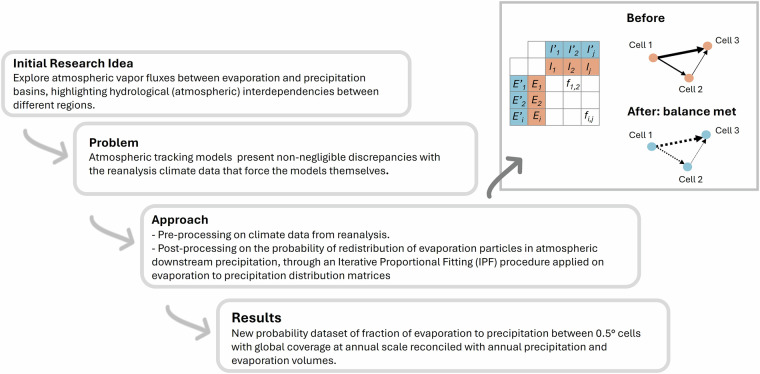
Schematic overview of the study and assay design.

To address this gap, this study proposes a reconciliation framework based on the Iterative Proportional Fitting (IPF) procedure^22–25^, a rigorous mathematical framework for refining tracking model outputs, thereby reducing uncertainties arising from modeling assumptions and data resolution constraints. This study further includes a pre-processing of the ERA5 reanalysis data to address the existing annual unbalance between ERA5 precipitation and evaporation^10^. Overall, the entire reconciliation framework ensures that the total tracked atmospheric moisture matches the total precipitation at the sink and the total evaporation at the source on an annual basis and in each cell. Figure 1 illustrates the steps of the framework, developed to reconcile these discrepancies and accurately explore atmospheric vapour flows between evaporation and precipitation, highlighting the hydrological interdependencies between different regions.

The outcome is a new dataset of moisture flow volumes from sources of evaporation to fates of precipitation at 0.5°, with global coverage and centred over 2008-2017, which aligns coherently with annual precipitation and evaporation volumes from ERA5 reanalysis. The reconciled cell-grid dataset provided here offers post-processed atmospheric moisture portions of evaporation at the source precipitating at the sink which closes the atmospheric hydrological balance on the annual basis. This marks a significant advancement in enhancing the reliability of the UTrack dataset and paves the way for future applications across multiple tracking models and forcing data.

## Methods

### Data

The atmospheric moisture connection data are sourced from the UTrack dataset^14^ – openly available at 10.1594/PANGAEA.912710. The UTrack dataset is available for a reference average year *y* centred on the period 2008-2017, on a monthly basis (*m*) and at cell-scale resolutions of 0.5° and 1°. In the dataset, the selection of a source cell *s* (identified through the location of evaporation) gives a global matrix of the monthly forward footprint, *pf*(*s*, *t*, *m*), of atmospheric moisture (i.e., the fraction of evaporation from the selected cell *s* that reaches each sink cell *t*, in the month *m*). The dataset is based on the Lagrangian atmospheric moisture tracking model UTrack^3^, forced with ERA5 hourly atmospheric moisture content for 25 atmospheric layers in the troposphere at 0.25° horizontal resolution (Copernicus Climate Change Service, C3S)^14^.

Thus, to reconstruct the moisture flows from the UTrack probability dataset and to apply the IPF procedure, climatic data of precipitation and evaporation are sourced from ERA5 reanalysis on single levels from 2008 to 2017 provided by the ECMWF Climate Data Store (Copernicus Climate Change Service, C3S). The monthly-averaged data of precipitation and evaporation at 0.25° in the cell *c* for each year *y* from 2008 to 2017, namely *P*_*ERA*5_(*c*, *m*, *y*) and *ET*_*ERA*5_(*c*, *m*, *y*), expressed in meter per day, are re-gridded at 0.5° with bilinear interpolation through the CDO operator *remapbil* on a grid [(90,−90),(0,360)] for consistency with the UTrack dataset, available at 0.5° spatial resolution. We calculate the area of the generic cell *c* grid *a*(*c*) through the *gridarea* operator from the Climate Data Operators (CDO) software, a collection of many operators for standard processing of climate and forecast model data^26^. The reference grid to calculate the area of each cell is the input data from the UTrack dataset at the spatial resolution of 0.5°.

Regarding the physics of the UTrack model^3^, the movement of each parcel is determined by three-dimensional wind patterns. With each time step, the wind pushes the parcel, creating a path (trajectory) that shows where the moisture travels. Once precipitation occurs at the parcel’s location, some of its moisture is allocated to precipitation at the corresponding ERA5 grid cell. The fraction of moisture that is allocated is determined by the ratio of the local precipitation (*P*) at that time step over the total amount of water in the atmospheric column (*P*_*w*_) according to the ERA5 data. As the parcel with moisture continues moving, more of it rains out at different locations. The process stops when either 99% of the original moisture in the parcel has rained out or 30 days have passed. In other words, any source location has a spatially distributed sink, which is determined by all the trajectories of moisture parcels released from the source location and by the precipitation events that happened along these trajectories. After a part of the tracked moisture precipitates along the parcel trajectory, it may indeed re-evaporate. As such, this evaporation is considered a new source, which again results in a distributed sink associated with the trajectories of the moisture parcels that represent the respective evaporation. It can be argued that indirect source-sink relations via intermediate precipitation and re-evaporation also constitute a source-sink connection. However, these indirect connections, which have also been called “cascading moisture recycling”^27,28^, are not explicitly included in the dataset, as counting the same amount of moisture twice if it re-evaporates and re-precipitates would violate the conservation of mass. We refer to the original study^3^ for an in-depth description of the model assumptions.

### Processing of ERA5 precipitation and evaporation data

Since UTrack data are given as a ten-year average between 2008 and 2017, we average ERA5 precipitation *P*_*ERA*5_(*c*, *m*, *y*) and evaporation *ET*_*ERA*5_(*c*, *m*, *y*) reanalysis data on the same ten-year period, as:

1 $${\widehat{P}}_{ERA5}(c,m)=\frac{1}{10}\cdot \mathop{\sum }\limits_{y=2008}^{2017}{P}_{ERA5}(c,m,y)\qquad [{\rm{m}}\,{{\rm{day}}}^{-1}]$$

2 $${\widehat{ET}}_{ERA5}(c,m)=\frac{1}{10}\cdot \mathop{\sum }\limits_{y=2008}^{2017}E{T}_{ERA5}(c,m,y)\qquad [{\rm{m}}\,{{\rm{day}}}^{-1}]$$

The reconciliation procedure is based on annual volumes, therefore the monthly ERA5 precipitation and evaporation data from the Climate Data Store (CDS) – ${\widehat{P}}_{ERA5}(c,m)$ and ${\widehat{ET}}_{ERA5}(c,m)$ – expressed in meters per day are multiplied for the length of the month and for the area of each cell to get the total precipitated or evaporated volume in the month *m* at the cell *c*:

3 $${\widehat{P}}_{ERA5}(c,m)={\widehat{P}}_{ERA5}(c,m)\cdot a(c)\cdot d(m)\qquad [{{\rm{m}}}^{3}{{\rm{month}}}^{-1}]$$

4 $${\widehat{ET}}_{ERA5}(c,m)={\widehat{ET}}_{ERA5}(c,m)\cdot a(c)\cdot d(m)\qquad [{{\rm{m}}}^{3}{{\rm{month}}}^{-1}]$$

where *a*(*c*) is the area of the cell in squared meters and *d*(*m*) are the days per month.

#### Handling ERA5 condensation values

Evaporation in the ERA5 hourly dataset on single levels represents the total amount of water evaporating from the Earth’s surface into the atmosphere. This dataset accounts for both condensation (downward fluxes) and evaporation (upward fluxes), distinguished by their sign in the ECMWF Integrated Forecasting System (IFS): downward fluxes (condensation) are positive, while upward fluxes (evaporation) are negative. In this study, we reverse this convention, defining upward fluxes as positive (indicating evaporation) and downward fluxes as negative (indicating condensation), to be consistent with the UTrack dataset. In examining the ERA5 monthly evaporation data, we observe several grid cells at high latitudes in the Northern Hemisphere that exhibit negative values (condensation), which indicates where monthly condensation exceeds evaporation at least once per year (see Techincal Validation).On an annual basis, cells with negative values are mostly concentrated in Greenland and Antarctica (see Techincal Validation) where condensation exceeds evaporation, yielding a negative sum for ${\sum }_{m}{\widehat{ET}}_{{\rm{ERA5}}}(c,m)$.

To prepare the data for analysis, cells with slightly negative monthly evaporation values ${\widehat{ET}}_{{\rm{ERA5}}}(c,m)$ are adjusted to 10^−5^ m^3^month^−1^. This value serves as a proxy for null evaporation, ensuring the functionality of our processing framework, which requires non-zero values for operation. Although this assumption is not physically based, it has minimal impact on results elsewhere: condensation values are typically as small as 7  ⋅  10^−7^ m^3^ on average, and reach a maximum of 1  ⋅  10^−5^ m^3^, while evaporation values average around 2  ⋅  10^9^ m^3^ and and can reach up to about 10  ⋅  10^9^ m^3^.

#### Closing ERA5 global balance between precipitation and evaporation 2008-2017

On a global scale, total annual precipitation is expected to equal total annual evaporation. ERA5 balance between precipitation and evaporation is relatively good for a twenty-year period from the mid-1990s^10^, yet the annual balance is not well closed in more recent years (2013-2017)^10,29^. We refer to the analysis in De Petrillo *et al*.^29^ for a more in-depth illustration of the yearly difference between the ERA5 global precipitation and evaporation over the period 2008-2017.

Annual volumes ${\widehat{P}}_{ERA5}(c)$ and ${\widehat{ET}}_{ERA5}(c)$ for each cell *c* are calculated cumulating the monthly values as:

5 $${\widehat{P}}_{ERA5}(c)=\mathop{\sum }\limits_{m=1}^{12}{\widehat{P}}_{ERA5}(c,m)\qquad [{{\rm{m}}}^{3}{{\rm{yr}}}^{-1}]$$

6 $${\widehat{ET}}_{ERA5}(c)=\mathop{\sum }\limits_{m=1}^{12}{\widehat{ET}}_{ERA5}(c,m)\qquad [{{\rm{m}}}^{3}{{\rm{yr}}}^{-1}]$$

Then, global volumes of precipitation ${\widehat{P}}_{ERA5,g}$ and evaporation ${\widehat{ET}}_{ERA5,g}$ for the average year between 2008 and 2017 at the cell-scale:

7 $${\widehat{P}}_{ERA5,g}=\mathop{\sum }\limits_{c=1}^{{N}_{c}}{\widehat{P}}_{ERA5}(c)\qquad [{{\rm{m}}}^{3}{{\rm{yr}}}^{-1}]$$

8 $${\widehat{ET}}_{ERA5,g}=\mathop{\sum }\limits_{c=1}^{{N}_{c}}{\widehat{ET}}_{ERA5}(c)\qquad [{{\rm{m}}}^{3}{{\rm{yr}}}^{-1}]$$

where *N*_*c*_ is the total number of cells, namely 259200.

To respect the global hydrological balance, ${\widehat{P}}_{ERA5,g}$ and ${\widehat{ET}}_{ERA5,g}$ are imposed to equal their average (5.50  ⋅ 10^5^ km^3^ yr^−1^) and scaling factors *α*_*P*_ and *α*_*ET*_ are obtained as:

9 $${\alpha }_{ET}=\frac{{\widehat{P}}_{ERA5,g}+{\widehat{ET}}_{ERA5,g}}{2}\cdot \frac{1}{{\widehat{ET}}_{ERA5,g}}\qquad {\rm{[-]}}$$

10 $${\alpha }_{P}=\frac{{\widehat{P}}_{ERA5,g}+{\widehat{ET}}_{ERA5,g}}{2}\cdot \frac{1}{{\widehat{P}}_{ERA5,g}}\qquad {\rm{[-]}}$$

Scaling factors obtained are *α*_*P*_ = 0.9971 and *α*_*ET*_ = 1.0027. These values slightly differ from the ones found in De Petrillo *et al*.^29^ where the same approach is used, since in the country/ocean scale study, negative values of evaporation indicating condensation are assimilated in the aggregation at the country/ocean scale and thus do not recur as negative values in the aggregated matrix.

*α*_*P*_ and *α*_*ET*_ are used here to re-scale the monthly precipitation ${\widehat{P}}_{ERA5}(c,m)$ volumes and evaporation ${\widehat{ET}}_{ERA5}(c,m)$ volumes in the average year, as:

11 $${\overline{\widehat{P}}}_{ERA5}(c,m)={\alpha }_{P}\cdot {\widehat{P}}_{ERA5}(c,m)\qquad [{{\rm{m}}}^{3}{{\rm{month}}}^{-1}]$$

12 $${\overline{\widehat{ET}}}_{ERA5}(c,m)={\alpha }_{ET}\cdot {\widehat{ET}}_{ERA5}(c,m)\qquad [{{\rm{m}}}^{3}{{\rm{month}}}^{-1}]$$

where ${\overline{\widehat{P}}}_{ERA5}(c,m)$ and ${\overline{\widehat{ET}}}_{ERA5}(c,m)$ are the corrected ERA5 monthly precipitation and evaporation fields for the average year between 2008 and 2017.

The monthly values are then summed over the year to obtain annual volumes of adjusted precipitation and evaporation for each cell *c*, namely ${\overline{\widehat{P}}}_{ERA5}(c)$ and ${\overline{\widehat{ET}}}_{ERA5}(c)$ as:

13 $${\overline{\widehat{P}}}_{ERA5}(c)=\mathop{\sum }\limits_{m=1}^{12}{\overline{\widehat{P}}}_{ERA5}(c,m)\qquad [{{\rm{m}}}^{3}{{\rm{yr}}}^{-1}]$$

14 $${\overline{\widehat{ET}}}_{ERA5}(c)=\mathop{\sum }\limits_{m=1}^{12}{\overline{\widehat{ET}}}_{ERA5}(c,m)\qquad [{{\rm{m}}}^{3}{{\rm{yr}}}^{-1}]$$

### UTrack moisture flows reconstruction

In the UTrack dataset, the selection of cell *c* (identified through the location of evaporation) gives a global matrix of precipitation shed (i.e., the downwind ocean and land surface receiving precipitation from evaporation in *c*). Hereafter the generic cell *c* is referred to as *s* when it acts as a source cell (contributing evaporation to downwind areas’ precipitation), and *t* when it acts as a sink cell (receiving precipitation from upwind areas’ evaporation). Specifically, we define **S** as the space encompassing all possible source worlds where evaporation contributes to precipitation downwind and **T** as the space of all sink worlds receiving precipitation sourceating from upwind evaporation. Each specific source world *S* ∈ **S** and sink world *T* ∈ **T** have dimensions 360 × 720, representing global matrices of potential source and sink cells, respectively. In this framework, a generic source cell *s* = (*lon*_*s*_, *lat*_*s*_) ∈ *S* is any cell within a source world S, and a sink cell *t* = (*lon*_*t*_, *lat*_*t*_) ∈ *T* is any cell within a sink world T.

#### Flow reconstruction from sources of evaporation to sinks of precipitation

Let *T*(*s*) ∈ **T** denote the sink world associated with a specific source cell *s* ∈ *S*. From the partition of evaporation from a cell *s* ∈ *S* to a sink world *T*(*s*), namely *pf*(*s*, *t*, *m*) extracted from the UTrack dataset (see Section Data), the monthly atmospheric moisture flow *ff*(*s*, *t*, *m*), is evaluated as:

15 $$ff(s,t,m)={\overline{\widehat{ET}}}_{ERA5}(s,m)\cdot pf(s,t,m)\cdot \qquad \forall s\in S,\forall t\in T(s)\qquad [{{\rm{m}}}^{3}{{\rm{month}}}^{-1}]$$

where ${\overline{\widehat{ET}}}_{ERA5}(s,m)$ is defined as in Equation (12).

#### Flow reconstruction from sinks of precipitation to sources of evaporation

Let *S*(*t*) ∈ *S* denote the source world associated with a specific sink cell *t* ∈ *T*. From the partition of precipitation in a sink cell *t* into its evaporation source cells *s* ∈ *S*(*t*), namely *pb*(*s*, *t*, *m*) extracted from the UTrack dataset (see Section Data), the monthly atmospheric moisture flow *fb*(*s*, *t*, *m*), is evaluated as:

16 $$fb(s,t,m)={\overline{\widehat{P}}}_{ERA5}(s,m)\cdot pb(s,t,m)\qquad \forall t\in T,\forall s\in S(t)\qquad [{{\rm{m}}}^{3}{{\rm{month}}}^{-1}]$$

where ${\overline{\widehat{P}}}_{ERA5}(s,m)$ is defined as in Equation (11).

#### Yearly moisture flows centered on 2008-2017

At this point, the monthly bilateral moisture forward flows *ff*(*s*, *t*, *m*) and backward flows *fb*(*s*, *t*, *m*) are summed all over the year, as follows:

17 $$ff(s,t)=\mathop{\sum }\limits_{m=1}^{12}ff(s,t,m)\qquad [{{\rm{m}}}^{3}{{\rm{yr}}}^{-1}]$$

18 $$fb(s,t)=\mathop{\sum }\limits_{m=1}^{12}fb(s,t,m)\qquad [{{\rm{m}}}^{3}{{\rm{yr}}}^{-1}]$$

If summing all the contributing evaporation sheds from the world of sources *S* to a sink *t* (source-to-sink reconstructed flows *ff*(*s*, *t*)), we obtain the total reconstructed annual precipitation in *t* from contributions of evaporation from all *s* ∈  *S*, namely:

19 $${P}_{reconstructed}(t)=\sum _{s}ff(s,t)$$

and viceversa, if summing all the contributing precipitation sheds from the world of sink *T* to a source *s* (sink-to-source reconstructed flows *fb*(*s*, *t*)), we obtain the total reconstructed annual evaporation in *s* from fractions of precipitation from all *t* ∈  *T*, namely:

20 $$E{T}_{reconstructed}(s)=\sum _{t}fb(s,t)$$

Comparing *P*_*reconstructured*_(*t*) and *ET*_*reconstructured*_(*s*) to forcing data ${\overline{\widehat{P}}}_{ERA5}(t)$ and ${\overline{\widehat{ET}}}_{ERA5}(s)$ it emerges that a deviation between the UTrack reconstructed annual tracked flows and the underlying forcing data from ERA5 reanalysis exists, namely:

21 $${\overline{\widehat{P}}}_{ERA5}(t)\ne {P}_{reconstructed}(t)$$

and:

22 $${\overline{\widehat{ET}}}_{ERA5}(s)\ne E{T}_{reconstructed}(s)$$

**Fig. 2:**
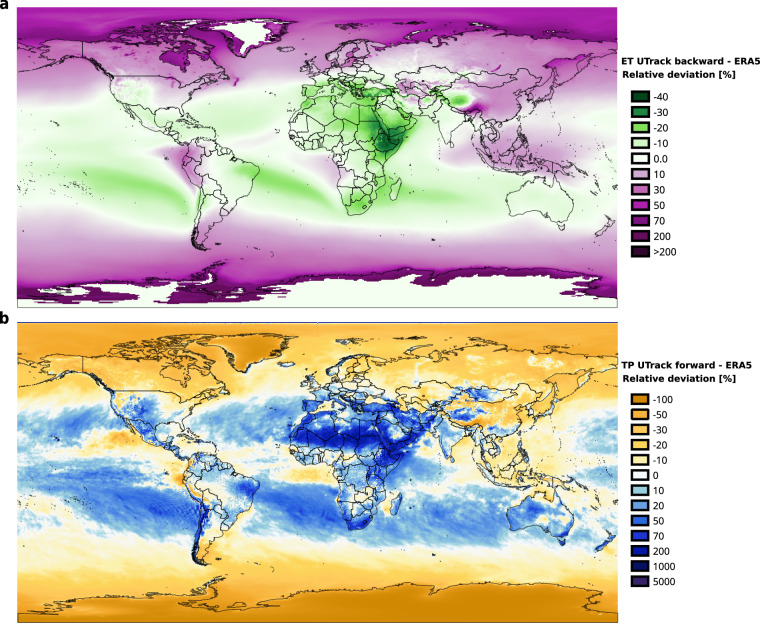
Percentage relative differences between reconstructed flows of (**a**) evaporation at the source in the sink-to-source (backward) reconstruction and (**b**) precipitation at the sink in the source-to-sink (forward) reconstruction, compared to the adjusted annual evaporation and precipitation flows from ERA5, respectively. In panel **a**, condensation values are excluded from both UTrack reconstructed flows and ERA5 data, resulting in a null deviation.

The discrepancies in Equation (22) and Equation (21) are shown in Fig. 2 (panel a and b respectively). To correct them, we separately apply an iterative proportional fitting (IPF) procedure and bi-proportionally adjust the source-to-sink and sink-to-source reconstructed flows, re-scaling the rows and the columns by the minimum amount necessary, to respect the sum constraints ${\overline{\widehat{ET}}}_{ERA5}(s)$ and ${\overline{\widehat{P}}}_{ERA5}(t)$ until they converge toward a balanced matrix^24,30^.

### Iterative Proportional Fitting Procedure

The iterative proportional fitting (IPF) procedure is a bi-proportional iterative adjustment that re-scales the elements of a matrix, e.g., *ff*(*s*, *t*), by estimating a new matrix $\overline{ff(s,t)}={\alpha }_{s}{\alpha }_{t}ff(s,t)$ at each iteration *η*, until convergence is found within a pre-defined tolerance^22,24,30^ and such that the marginals of the matrix meet the following conditions:

23 $${P}_{reconstructed}(t)={\overline{\widehat{P}}}_{ERA5}(t)\quad \,{\rm{and}}\,\quad E{T}_{reconstructed}(s)={\overline{\widehat{ET}}}_{ERA5}(s)$$

In our case *ff*(*s*, *t*) is the reconstructed matrix when referring to the source-to-sink reconstruction, or *fb*(*s*, *t*) when referring to the sink-to-source reconstruction – and the marginals are the ERA5 corrected data of evaporation ${\overline{\widehat{ET}}}_{{\rm{ERA5}}}(s)\,\forall s\in S$ and precipitation ${\overline{\widehat{P}}}_{{\rm{ERA5}}}(t)\,\forall t\in T$. In this problem, each iteration *η*, to satisfy Equation (23), involves correcting *n*^2^ cells with *n* correction factors, having *n* = 259200. Indeed, each source cell *s* ∈ *S* (location of evaporation) is associated with a global matrix *T*(*s*) of dimensions (360 × 720) of tracked moisture flows *ff*(*s*, *t*) (i.e., the volume of evaporation from the selected cell *s* precipitating at each sink cell *t* in the world *T*(*s*)) calculated as in Equation (15), and vice-versa for the sink-to-source reconstructed moisture flow *fb*(*s*, *t*) calculated as in Equation (16).

Extending on the IPF application on a 2D moisture flow matrix^29^, here we expand the IPF application to a multidimensional setting of atmospheric moisture connections. Different studies deal with various applications of bilateral iterative adjustment as multidimensional problems, for example by vectorising a multidimensional representation into a one-to-one correspondence with a unidimensional vector^31–33^ or by adopting various optimisation functions, including parallelisation techniques and Cimmino algorithms^34,35^.

Some implementation packages, namely the R package mipfp^36^ and an equivalent Python package, ipfn^37^, have been developed to provide a multi-dimensional implementation of the traditional IPF procedure - allowing the updating of an N-dimensional array for given sink marginal distributions. However, in this problem, we found these packages to require a heavy computation effort since they do not allow parallel programming in the logical structure designed for this study. Thus, our approach implements a different strategy to avoid an excessive computational burden and the impossibility of using parallel programming. Our approach involves parallelization and vectorization to establish a one-to-one correspondence between all sources *s* ∈ *S* and their respective sinks *t* ∈ *T*(*s*). This method is specifically designed to manage the multidimensional nature of bilateral moisture connections between the source space **S** and the sink space **T**.

For the sake of simplicity, we present here the IPF procedure on the source-to-sink reconstructed flows $\overline{ff}(s,t)\,\forall s\in S,\forall t\in T(s)$. The same sequence of passages is valid for the sink-to-source reconstructed flows *fb*(*s*, *t*)  ∀*t* ∈ *T*, ∀*s* ∈ *S*(*t*).

The ad-hoc IPF procedure alternatively evaluates the adjusting factor *α*(*t*) for the reconstructed precipitation at the sink *t* ∈ *T*(*s*),  ∀*t* ∈ *T* and the adjusting factor *α*(*s*) for the reconstructed evaporation at the source *s*,  ∀*s* ∈ *S*.

Therefore, the ad-hoc IPF procedure corrects precipitation volumes at odd iterations and evaporation volumes at even iterations. The procedure continues for several iterations until convergence is found. Emblematically, the first two iterations are described hereafter.

When *η* = 1, each moisture flow from a source cell *s* ∈ *S* to the associated sink world *T*(*s*) is adjusted to meet the precipitation volume  ∀ *t* ∈ *T*(*s*), namely $\overline{{\widehat{P}}_{ERA5}}(t)$, as:

24 $$\alpha {(t)}^{\eta =1}=\frac{{\overline{\widehat{P}}}_{ERA5}(t)}{{\sum }_{s{\prime} \in S}ff(s{\prime} ,t)}\qquad {\rm{[-]}}$$

The adjusted source-to-sink flow from the source *s* matching ERA5 precipitation  ∀*t* ∈ *T*(*s*) is then defined as:

25 $$ff{\prime} (s,t)=ff(s,t)\alpha {(t)}^{\eta =1}\quad \forall s\in S,\forall t\in T(s)\qquad [{{\rm{m}}}^{3}{{\rm{yr}}}^{-1}]$$

**Fig. 3:**
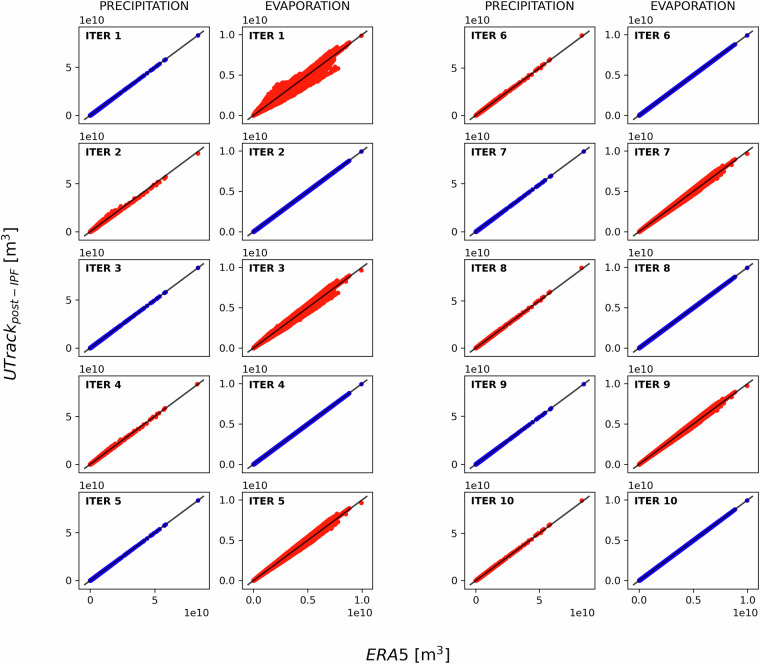
Scatter-plots comparing sum of all sink worlds (precipitation) and all source worlds (evaporation) from UTrack estimates post-IPF vs. ERA5, for iterations 1-10. Odd iterations illustrate the adjustment on precipitation in the sink worlds *T* ∈ **T** (in blue) and its corresponding perturbation of evaporation in the source worlds *S* ∈ **S** (in red). Vice-versa even iterations illustrate the adjustment on evaporation in the source worlds *S* ∈ **S** (in blue) and its corresponding perturbation of precipitation in the sink worlds *T* ∈ **T** (in red). By iteration 10, both precipitation and evaporation values exhibit a good fit to ERA5. These iterations refer to the source-to-sink reconstructed moisture flow matrix.

This correction creates an inconsistency with the evaporation from the space source worlds *S* ∈ **S**, as shown in the scatterplots in Fig. 3, therefore in the next iteration a correction factor to match evaporation constraints in the source worlds *S* ∈ **S** is calculated as:

26 $$\alpha {(s)}^{\eta =2}=\frac{{\overline{\widehat{ET}}}_{ERA5}(s)}{{\sum }_{t{\prime} \in T(s)}ff{\prime} (s,t{\prime} )}\qquad {\rm{[-]}}$$

and applied, for example at iteration *η* = 2, as:

27 $$\overline{ff(s,t)}=ff{\prime} (s,t)\alpha {(s)}^{\eta =2}=ff(s,t)\alpha {(t)}^{\eta =1}\alpha {(s)}^{\eta =2}\quad \forall s\in S,\forall t\in T(s)\qquad [{{\rm{m}}}^{3}{{\rm{yr}}}^{-1}]$$

**Fig. 4:**
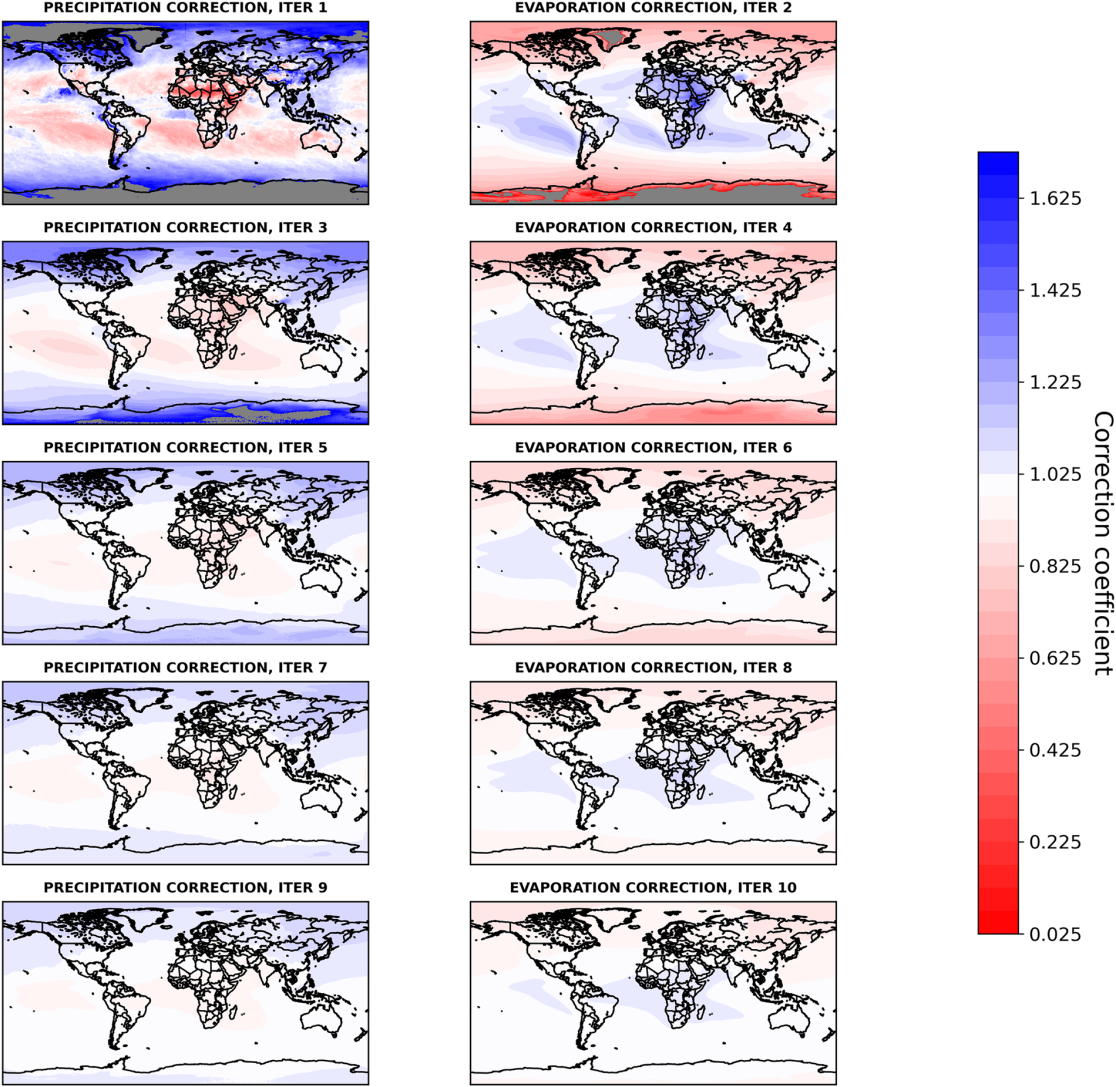
Geographical distributions of correction factors *α*(*t*) for precipitation (${\widehat{P}}_{UTrack}$) and *α*(*s*) for evaporation ${\widehat{ET}}_{UTrack}$ alternatively evaluated at odd and even iterations from 1-10 iterations, respectively, through the Iterative Proportional Fitting procedure. The divergent colour gradient indicates an incremental (blue) or decremental (red) adjustment of the estimated value of ${\widehat{P}}_{UTrack}$) or ${\widehat{ET}}_{UTrack}$. The color gradient varies around values close to 1. Cells adjusted with out-of-range coefficients (significantly exceeding 1) during the initial iterations are shown in grey. These iterations refer to the source-to-sink reconstructed moisture flow matrix.

Figure 3 illustrates well the inconsistency that each adjustment to precipitation in the sink worlds *T* ∈ **T** creates on evaporation in the source worlds *S* ∈ **S**. However, it also shows how rapidly these adjustments converge. By iteration 10, both precipitation and evaporation values exhibit a good fit. Another significant insight gained from analysing the iterative process is the geographical distribution of incremental (in blue) and decremental (in red) adjustments for precipitation (at odd iterations) and evaporation (at even iterations), as illustrated in Fig 4. By iteration 10, both the incremental and decremental factors begin to converge toward a neighbourhood of 1. When compared to Fig 2, Fig. 4 also illustrates the geographic distribution of the adjustments. Especially in the first iterations, these are particularly marked in regions with the highest deviations in Fig.2, namely the poles, arid areas, and oceans.

Finally, considering all iterations, the correction factors for precipitation at the sink C(t) and evaporation at the source R(s) can be expressed as:

28 $$R(s)=\prod _{\eta }\alpha {(s)}^{\eta }\quad {\rm{and}}\quad C(t)=\prod _{\eta }\alpha {(t)}^{\eta }$$

Hence, the generic adjusted bilateral moisture flow in the source-to-sink and sink-to-source reconstruction read, respectively:

29 $$\overline{ff(s,t)}=R(s)\cdot ff(s,t)\left.\right)\cdot C(i)\quad {\rm{and}}\quad \overline{fv(s,t)}=R(s)\cdot fb(s,t)\cdot C(i)$$

At this point, Equation (23) is satisfied and the discrepancies in Equation (21) and Equation (22) are solved.

**Fig. 5:**
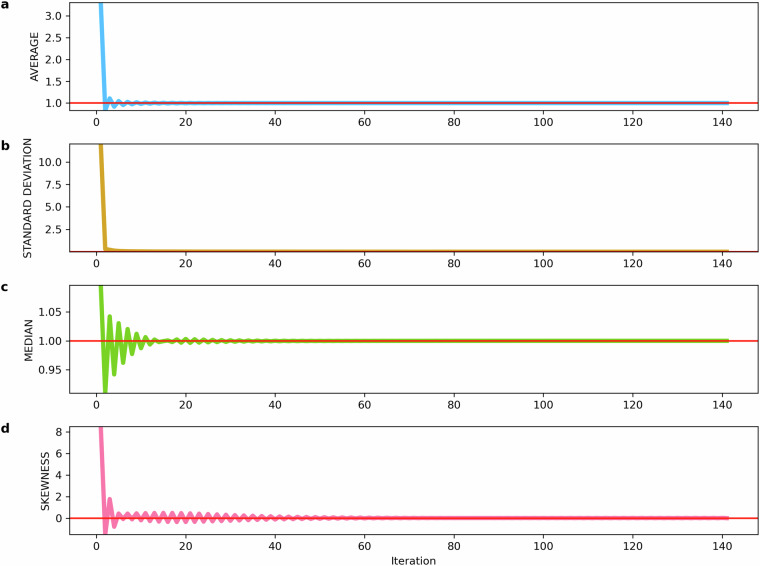
Statistics on (**a**) average, (**b**) standard deviation, (**c**) median, and (**d**) skewness of the distributions of alpha values *α*(*s*) and *α*(*t*), across each iteration of the Iterative Proportional Fitting (IPF) procedure for the source-to-sink reconstructed matrix flow. The fitting procedure continues until convergence is achieved, which occurs at the 140th iteration, with a convergence tolerance of 10^−5^.

The iterative process continues until all *α*(*t*) and *α*(*s*)  ∀*t* ∈ *T* and  ∀*s* ∈ *S* converge to a value in the neighbourhood of 1. In our problem, convergence is found for *η* = 140 and *η* = 139, in the source-to-sink and sink-to-source reconstruction, respectively, both cases within a tolerance of 10^−5^. At this point, Equation (23) is satisfied. Figure 5 shows the performance of the iterative process for the source-to-sink reconstructed flows *ff*(*s*, *t*) until convergence is satisfied, highlighting that a neighbourhood of convergence is already found after 15 iterations.

### Dataset building

The average reconciled flows $\overline{f(s,t)}$ are gathered in a 4D dataset of moisture volumes between sources of evaporation *s* and sinks of precipitation *t* for any world of sources *S* ∈ **S** and any world of sinks *T* ∈ **T**. To minimise the weight of the dataset, we convert floating point values to integers with the following procedure:

30 $$z=\left\{\begin{array}{l}0,\,{\rm{if}}\,y\le {y}_{\min }\\ {\rm{}}z=rint(1+\frac{lo{g}_{10}(y)-lo{g}_{10}({y}_{min})}{lo{g}_{10}({y}_{max})-lo{g}_{10}({y}_{min})}\cdot 254)\,{\rm{}},{\rm{if}}\,y > {y}_{\min }\end{array}\right.$$

where *z* is the converted a-dimensional moisture volume ranging from 0 to 255, *y* is the moisture volume, *y*_*max*_ ≃ 122079330 m^3^ yr − 1 is the maximum value of bilateral moisture volume flow $\overline{f(s,t)}$, *y*_*min*_ = 10^−3^ m^3^ yr − 1 is the lower threshold identifying consistent bilateral flows and the function rint is a Python function rounding floating point values to the nearest integer.

## Data Records

We release a dataset of moisture flow volumes (in cubic meters) from sources of evaporation to sinks of precipitation at 0.5° with global coverage and centred over 2008-2017, which aligns coherently with annual precipitation and evaporation volumes from ERA5 reanalysis, named RECON^38^. Data are collected in the *RECON*_*m*_*oisture*_*f*_*low*. *nc* file of moisture flows in cubic meters, obtained from the processing of the Lagrangian (forward trajectory-based) tracking model UTrack reconciled with ERA5 reanalysis through the reconciliation framework proposed in the study and based on the Iterative Proportional Fitting procedure. The file contains information on the generation of the dataset, authors of the dataset, and input variable information. The link to the archived dataset can be found at https://zenodo.org/records/14191920.

The structure of the RECON dataset is consistent with the UTrack dataset^39^, including the grid, latitude, and longitude ranges, while the variable of interest differs in units of measurements since the UTrack dataset provides a-dimensional partitioning of evaporation at the source contributing to precipitation at the sink (and vice-versa), the IPF-reconciled RECON dataset releases moisture connections between sources and sinks (and vice-versa) in cubic meters, to help users handle the process of retrieving from the uploaded dataset. The temporal scale of the dataset is annual.

Further description of the file structure and organisation of data within the dataset can be found in the “README" file in the online repository.

## Technical Validation

**Fig. 6:**
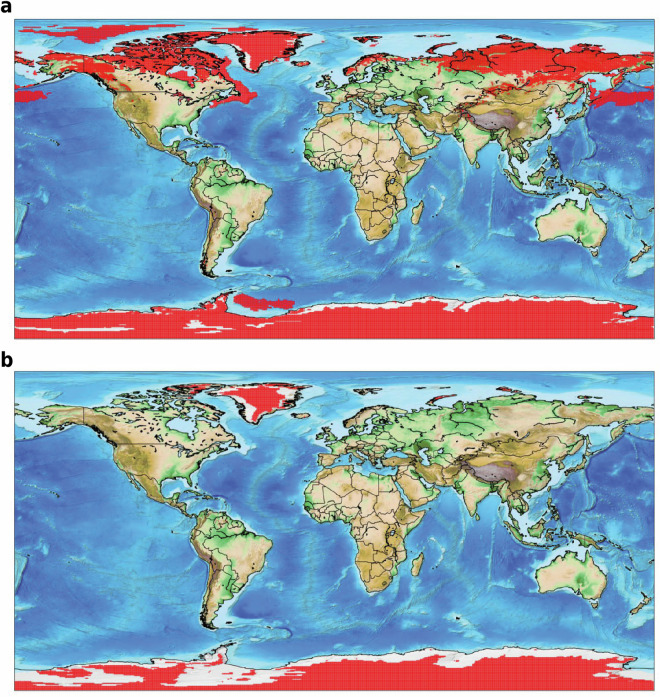
Negative values (indicating condensation) from the ERA5 dataset of evaporation on single levels, obtained from the Copernicus Climate Data Store^10^. (**a**) Cells where condensation occurs in at least one month of the average year between 2008 and 2017. (**b**) Cells where annual condensation exceeds evaporation in the average year between 2008 and 2017.

To validate the reconciliation procedure, an analysis of statistical significance is performed considering ten distinct randomly selected sub-samples. The selection pool for the sub-samples excludes points with negative initial evaporation values (Figure 6) which have been set to a value of 10^−5^, as these do not represent physical points within our study (see Section Processing of ERA5 precipitation and evaporation data).

**Fig. 7:**
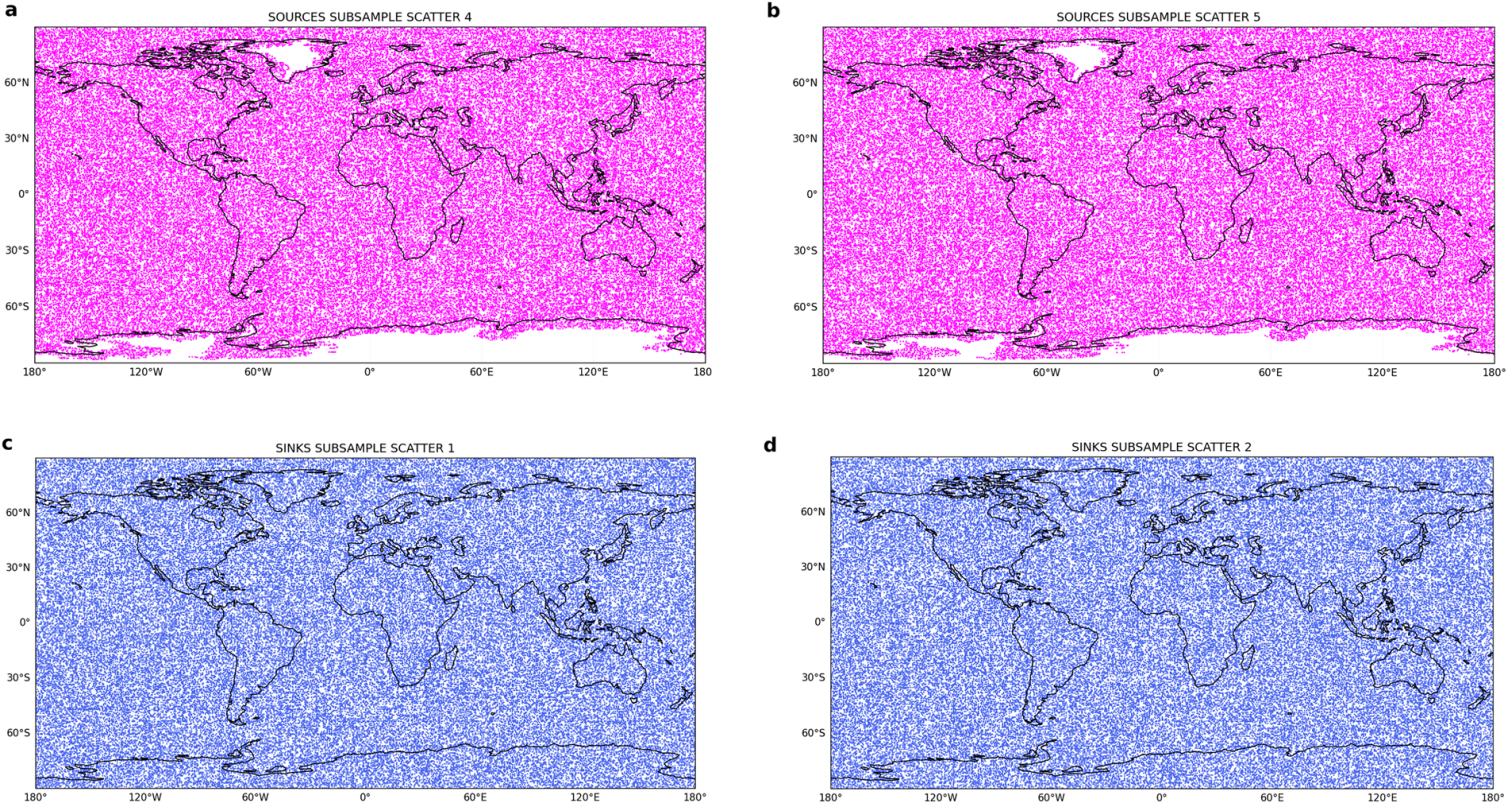
Locations of the 100’000 randomly selected points as sources (magenta) in **(a)** the sub-sample number 4, **(b)** number 5, and as sinks (blue) in the sub-sample **(b)** number 1 and **(**d) number 2.

The analysis is conducted, as illustrated in Fig. 7, for ten samples of 100’000 randomly selected points of sources *s* and sinks *t* – (lat_*s*_, lon_*s*_, lat_*t*_, lon_*t*_) – using a uniform distribution. The choice of sub-sampling is driven by the computational unfeasibility of statistically checking more than 67 billion points at once (made up by all sink worlds *T*(*s*) ∈ **T** associated with each source *s* ∈ **S**). However, ten sub-samples of 100’000 locations (out of a global total of 259,000 points) offer comprehensive global coverage of sources of evaporation and sinks of precipitation as comprehensively shown in Supplementary Figures 1–5 and Supplementary Figures 6–10. Figure 7 emblematically shows the randomly selected sources in the sub-samples 4 and 5 and the randomly selected sinks in the sub-samples 1 and 2.

**Fig. 8:**
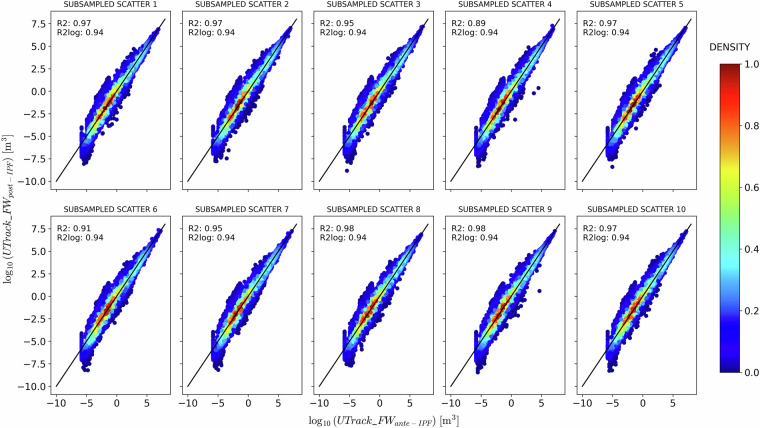
Comparison between *ante* − and *post* − IPF precipitation and evaporation estimates for ten samples of 100’000 randomly selected points of sources *s* and sinks *t* for the source-to-sink reconstructed flows.

**Fig. 9:**
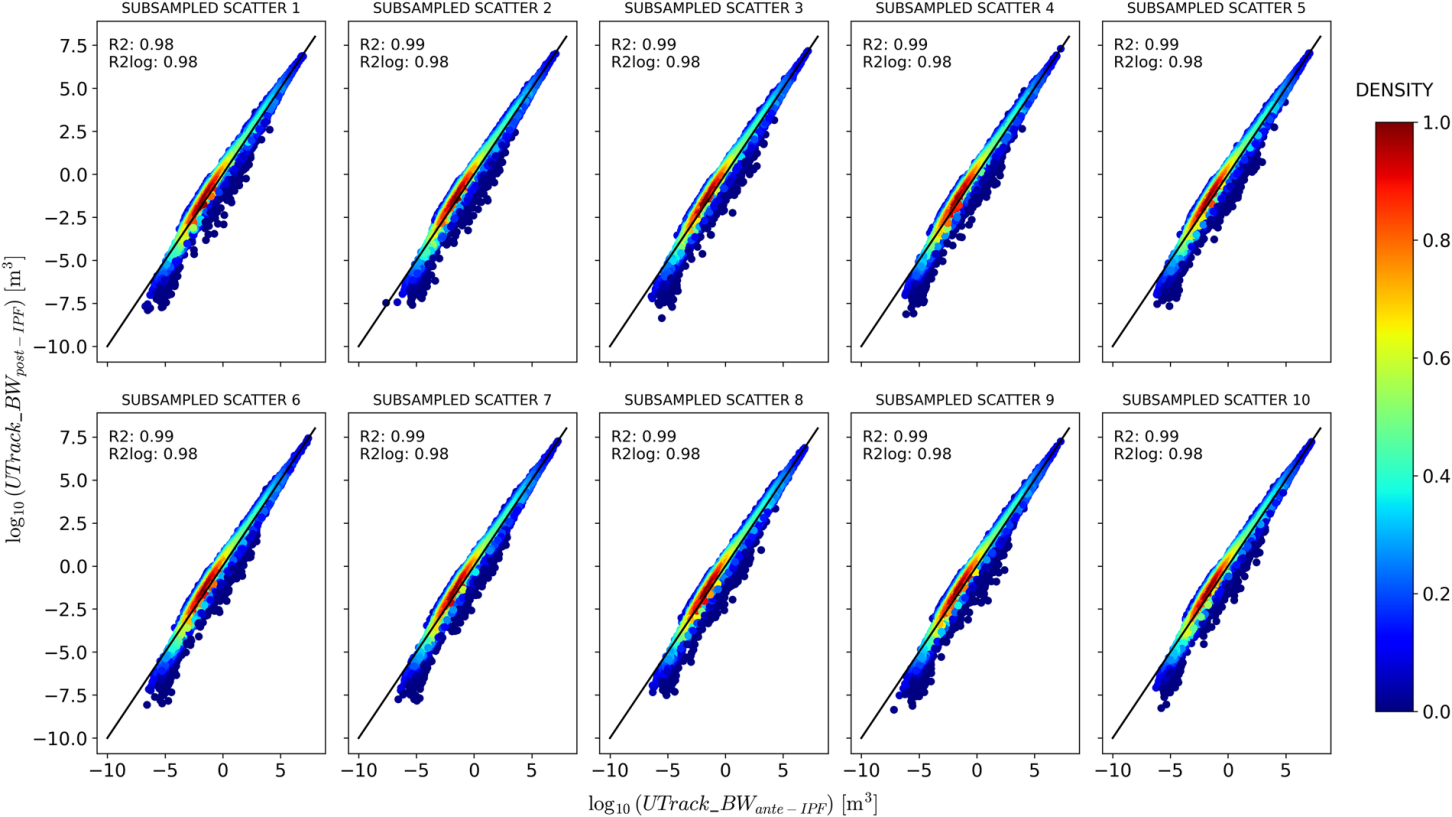
Comparison between *ante* − and *post* − IPF precipitation and evaporation estimates for ten samples of 100’000 randomly selected points of sources *s* and sinks *t* for the sink-to-source reconstructed flows.

Figures 8 and 9 compare precipitation estimates post-IPF against ante-IPF application on the source-to-sink and sink-to-source reconstructed flows, respectively.

A Gaussian kernel density estimation reveals a symmetric but broad distribution around the bisector for source-to-sink reconstructed flows (Fig. 8). In contrast, for sink-to-source reconstructed flows (Fig. 9), the distribution is more concentrated and less spread around the bisector, albeit less symmetrical, indicating a tendency for a flow reduction post-IPF. The density scatter plots in Figs. 8 and 9 demonstrate that the IPF adjustments preserve the structure of the atmospheric network modelled by the UTrack tracking. Specifically, points with a density greater than 0.6 closely follow the bisector line in both cases, as also supported by the high and similar *R*^2^ and ${R}_{\,\log }^{2}$ scores, which average approximately 0.97 and 0.94 in the source-to-sink case, and 0.99 and 0.98 in the sink-to-source one. These findings are consistent with the Iterative Proportional Fitting theory and corroborate results from De Petrillo *et al*.^29^, which indicate that IPF adjustment does not significantly impact the structure of bilateral connections.

While Figs. 8 and 9 aim at evaluating the flows’ changes in the bilateral connections ante- and post-IPF application in the source-to-sink and sink-to-source reconstructed matrices, they also show that although adjustments are not identical, the IPF performance in the two cases is comparable.

**Fig. 10:**
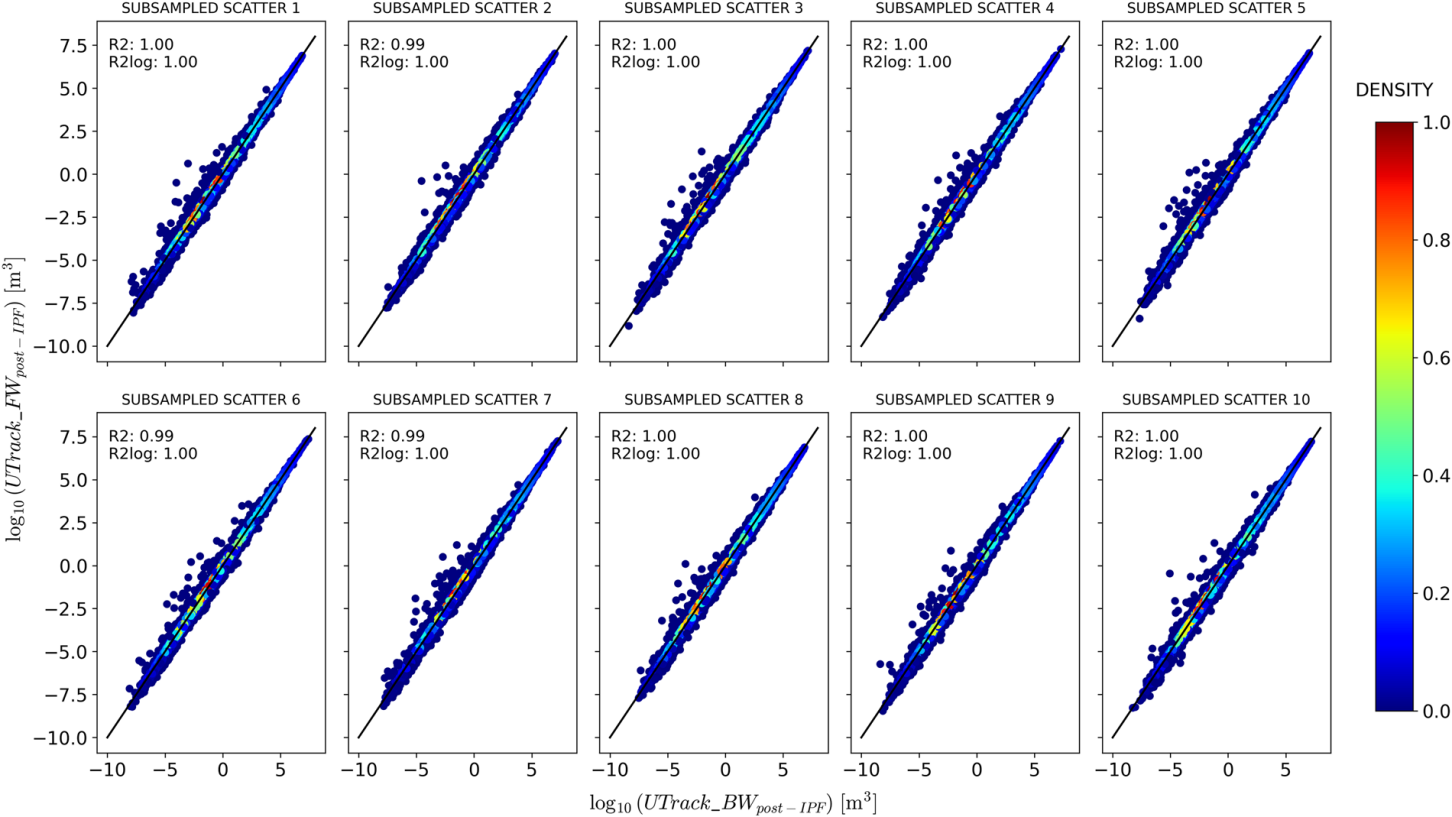
Comparison between post-IPF moisture flow estimates from sink-to-source reconstructed flows $\overline{fb(s,t)}$ on the x-axis and post-IPF source-to-sink reconstructed flows $\overline{ff(s,t)}$ on the y-axis, for ten samples of 100’000 randomly selected points of sources *s* and sinks *t*.

To further validate the IPF performance on the source-to-sink and sink-to-source reconstructions Figure 10 compares post-IPF moisture flow estimates from sink-to-source reconstructed flows and post-IPF source-to-sink reconstructed flows, for ten samples of 100’000 randomly selected points of sources and sinks. Results in Fig. 10 demonstrate on average a *R*^2^ = 1 indicating no difference between the source-to-sink and sink-to-source reconstructions in the performance of the IPF procedure. In light of this similar performance, to finalise the dataset, we average element-wise the post-IPF flows $\overline{ff(s,t)}$ and $\overline{fb(s,t)}$ and obtain a unique flow reconstruction $\overline{f(s,t)}$ between source and sink and vice-versa.

**Fig. 11:**
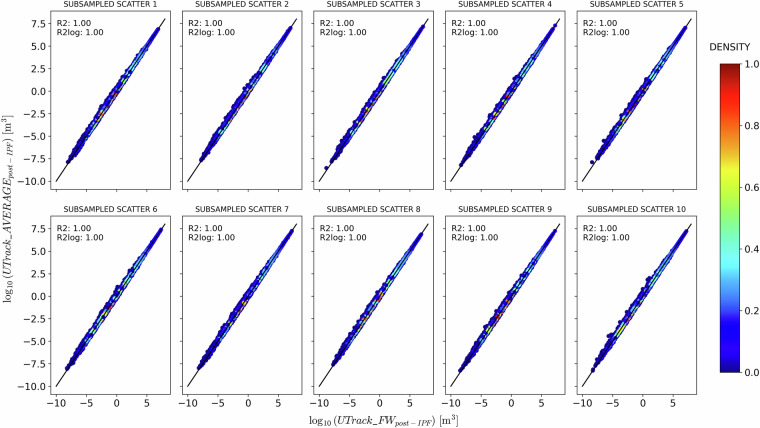
Comparison between post-IPF sink-to-source reconstructed flows $\overline{fb(s,t)}$ on the x-axis and the post-IPF flows $\overline{f(s,t)}$ obtained by averaging $\overline{fb(s,t)}$ with the post-IPF source-to-sink reconstructed flows$\overline{ff(s,t)}$ on the y-axis, for ten samples of 100’000 randomly selected points of sources *s* and sinks *t*.

**Fig. 12:**
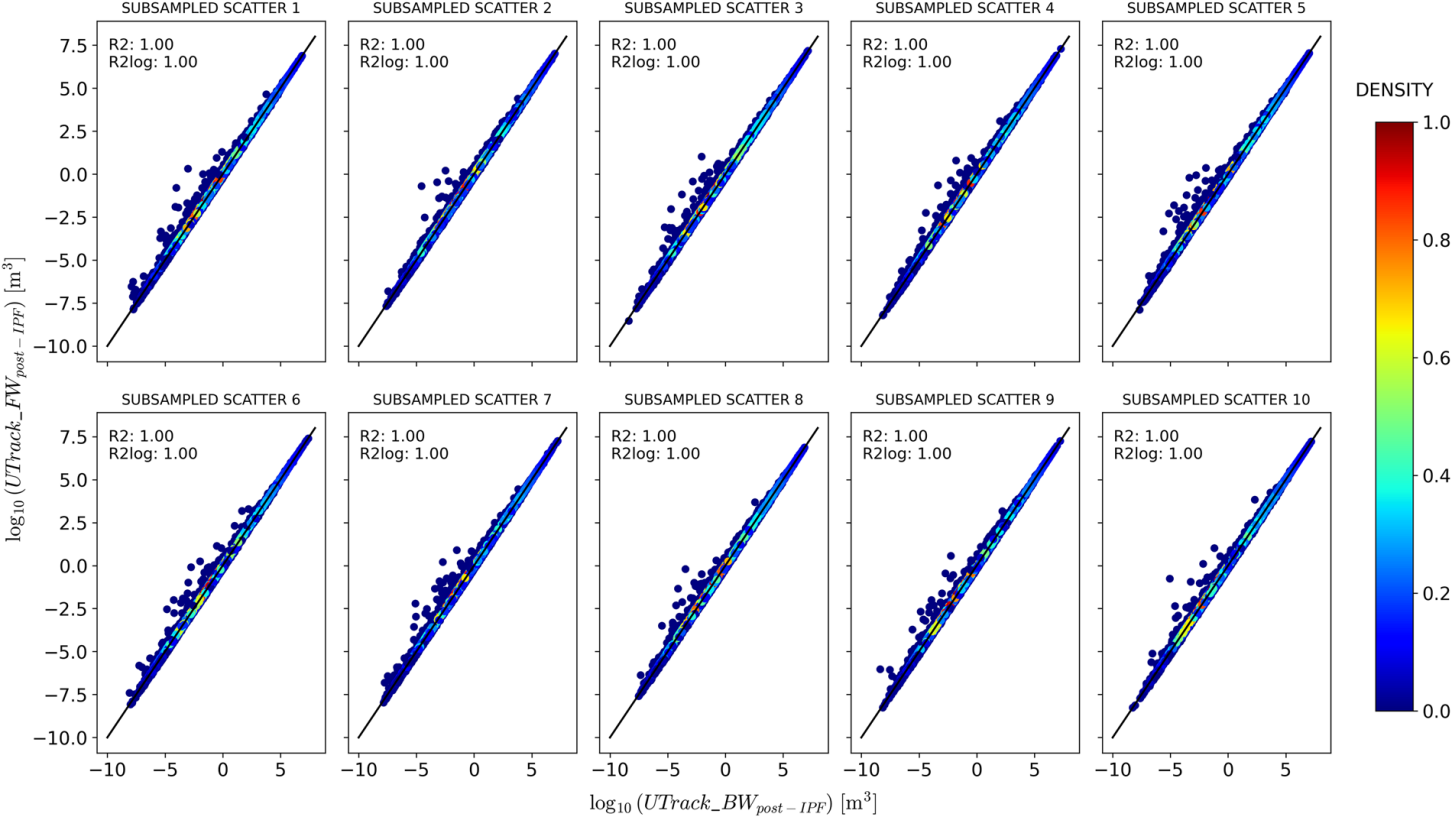
Comparison between post-IPF sink-to-source reconstructed flows $\overline{fb(s,t)}$ on the x-axis and the post-IPF flows $\overline{f(s,t)}$ obtained by averaging $\overline{fb(s,t)}$ with the post-IPF source-to-sink reconstructed flows$\overline{ff(s,t)}$ on the y-axis, for ten samples of 100’000 randomly selected points of sources *s* and sinks *t*.

To corroborate this choice, Fig. 11 and Fig. 12 compare the post-IPF bilateral flows in the source-to-sink ($\overline{ff(s,t)}$) and sink-to-source ($\overline{fb(s,t)}$) directions with their averaged matrix $\overline{f(s,t)}$. Specifically, Fig. 11 compares $\overline{ff(s,t)}$ with the average $\overline{f(s,t)}$, showing an excellent fit with the bisector line. In contrast, Fig. 12 illustrates the comparison between $\overline{fb(s,t)}$ and the average $\overline{f(s,t)}$, revealing a tendency to overestimate flows post-IPF. Despite this, both the *R*^2^ and ${R}_{log}^{2}$ values are in most parts indistinguishable from 1, indicating a high degree of fit.

The perfect fit observed in Fig. 11 and Fig. 12 substantiates the consistency of the IPF processing results, indicating that the averaged matrix reliably represents the final dataset.

Finally, we examine the alignment of the three IPF-reconciled matrices-$\overline{ff(s,t)}$, $\overline{fb(s,t)}$, and $\overline{f(s,t)}$-with ERA5 precipitation and evaporation (${\overline{\widehat{P}}}_{{\rm{ERA5}}}(c)$ and ${\overline{\widehat{ET}}}_{{\rm{ERA5}}}(c)$). This validation assesses the effectiveness of using averaged volumes to construct a dataset with a unique bilateral flow that represents atmospheric moisture connections between source *s* and sink *t*.

**Fig. 13:**
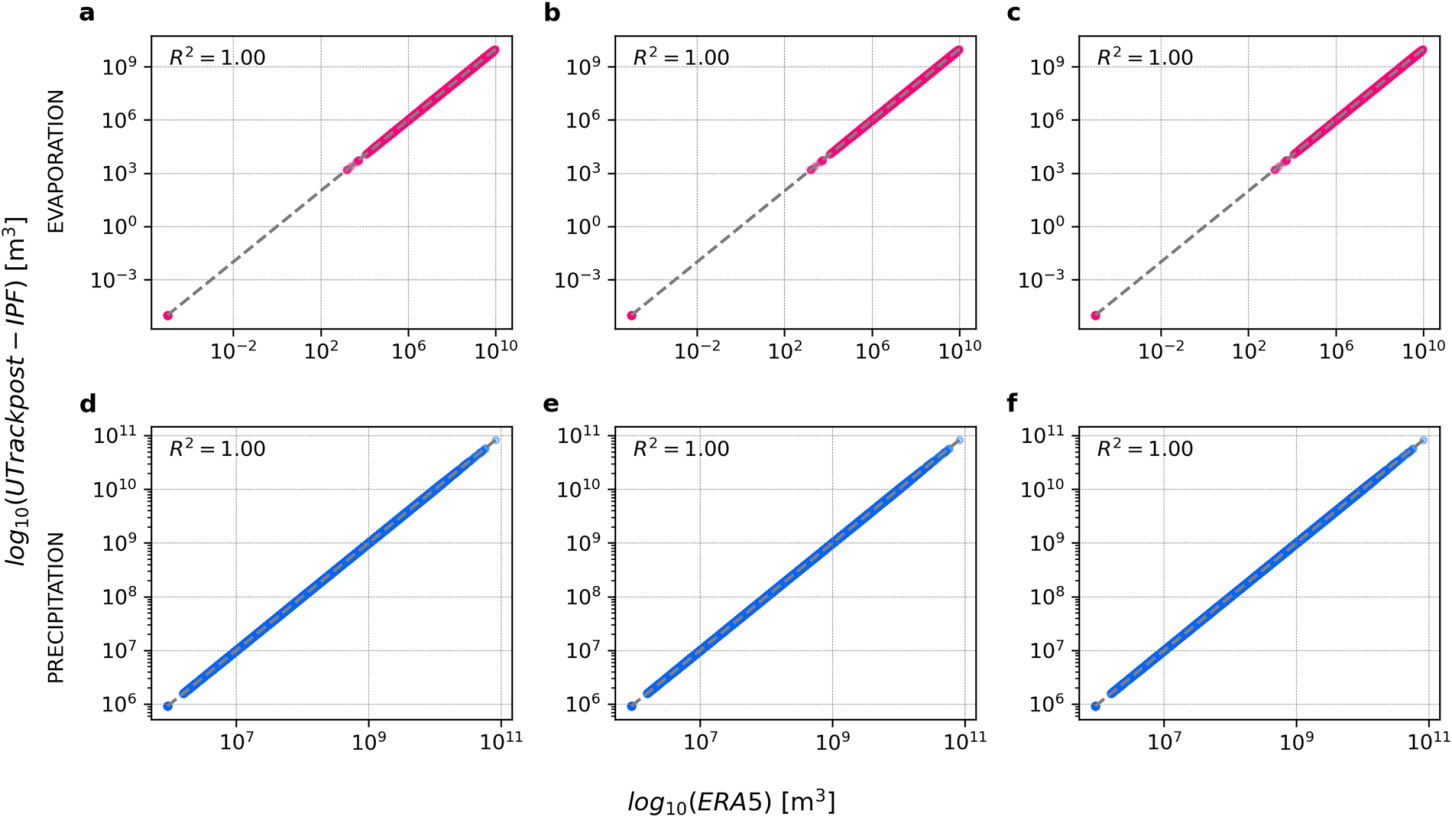
Fitness with ERA5 adjusted precipitation and evaporation annual values for the average year 2008-2017 of **a** post-IPF source-to-sink reconstructed flows$\overline{ff(s,t)}$, **b** post-IPF sink-to-source reconstructed flows $\overline{fb(s,t)}$, **c** post-IPF average flows $\overline{f(s,t)}$ for ten samples of 100’000 randomly selected points of sources *s* and sinks *t*.

Figure 13 illustrates that the annual precipitation and evaporation marginals for each source cell *s* and sink cell *t* perfectly match ERA5 precipitation and evaporation across all three cases. This alignment supports the choice of the average flow as the unique bilateral representation of atmospheric moisture connections, further corroborated by Figs. 11 and 12.

In conclusion, while the IPF procedure ensures that the marginals of the adjusted matrix align with total ERA5 annual evaporation and precipitation for all sources and sinks (Fig. 13), it does not compromise the physical integrity of the bilateral connections as shown in Figs. 8 and 9. Instead, it applies minimal corrections to each bilateral flow within the network, ensuring convergence while preserving the physical meaning of the reconstructed flows.

## Usage Notes

The conversion to retrieve annual moisture volumes in cubic meters from a-dimensional integer values ranging from 0 to 255 is the following:

31 $$y=1{0}^{\frac{z-1}{254}\cdot [lo{g}_{10}({y}_{max})-lo{g}_{10}({y}_{min})]+lo{g}_{10}({y}_{min})}$$

We refer to an in-depth description of all variables in Section Data Records.

To refine the extent of the shed, users can apply a filtering criterion in the conversion formula, excluding bilateral flows smaller than *y*_*min*_ according to their needs.

The data-processing workflow is available in the sample code at https://github.com/elenadepetrillo/RECON-globally-reconciled-moisture-flows

All the analyses were conducted within the Python environment (3.10). To handle the database users can use a Python environment with the required packages described in the GitHub repository https://github.com/elenadepetrillo/RECON-globally-reconciled-moisture-flows

## Supplementary information

Supplementary Information

## Data Availability

The codes developed for the building and processing of the data are available on GitHub at https://github.com/elenadepetrillo/RECON-globally-reconciled-moisture-flowsand on Zenodo at 10.5281/zenodo.14191920, respectively. Analyses were conducted within the Python environment (3.10) and required packages are indicated in the “requirements.txt” file on the GitHub repository.
