# Supplementary material for: Cell-scale atmospheric moisture flows dataset reconciled with ERA5 reanalysis
Source: Sci Data. 2025 Apr 15;12:629. doi: 10.1038/s41597-025-04964-3 (PMC12000399; doi:10.1038/s41597-025-04964-3)
Supplement: Supplementary file 1 — Supplementary Information [file 41597_2025_4964_MOESM1_ESM.pdf]

Supplementary Information

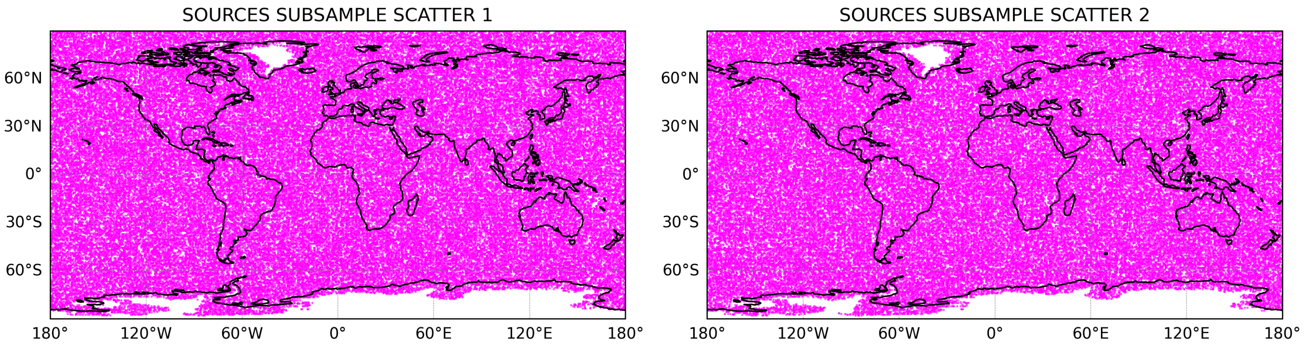

Supplementary Figure 1. Locations of the 100’000 randomly selected sources of evaporation in the first and second subsample.

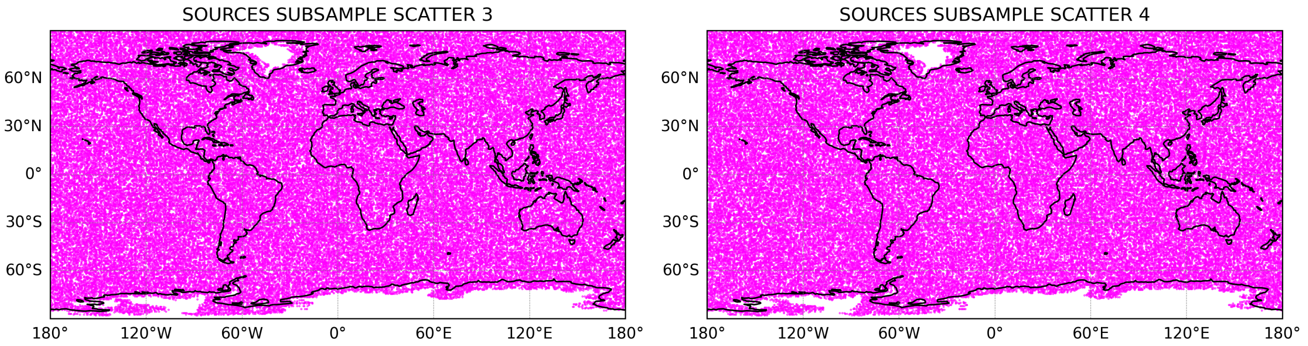

Supplementary Figure 2. Locations of the 100’000 randomly selected sources of evaporation in the third and fourth subsample.

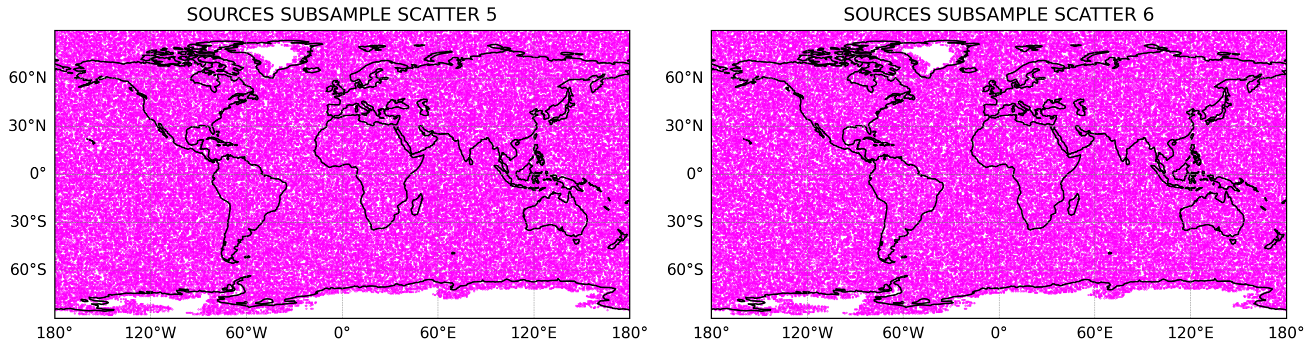

Supplementary Figure 3. Locations of the 100’000 randomly selected sources of evaporation in the fifth and sixth subsample.

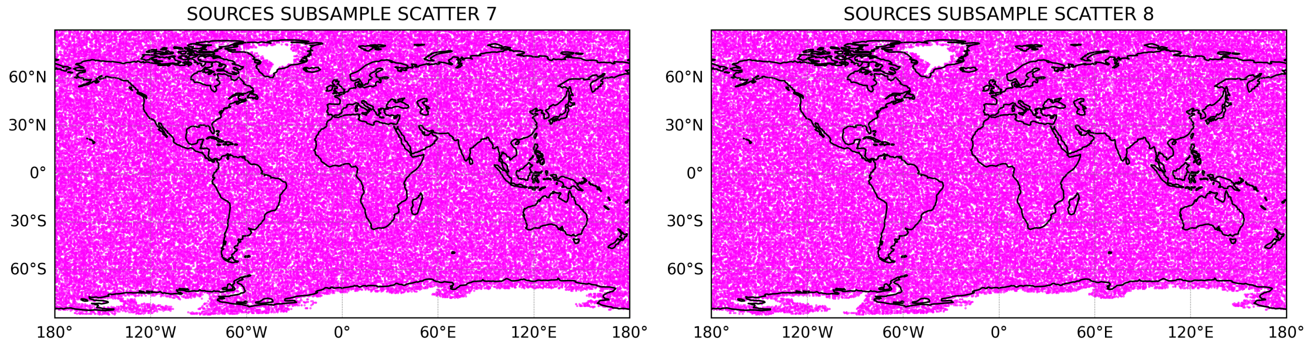

Supplementary Figure 4. Locations of the 100’000 randomly selected sources of evaporation in the seventh and eighth subsample.

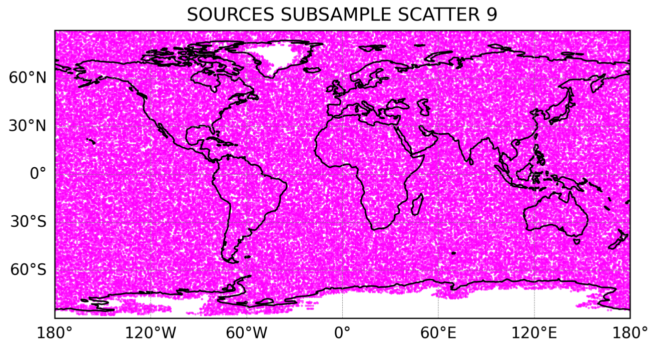

Supplementary Figure 5. Locations of the 100’000 randomly selected sources of evaporation in the ninth and tenth subsample.

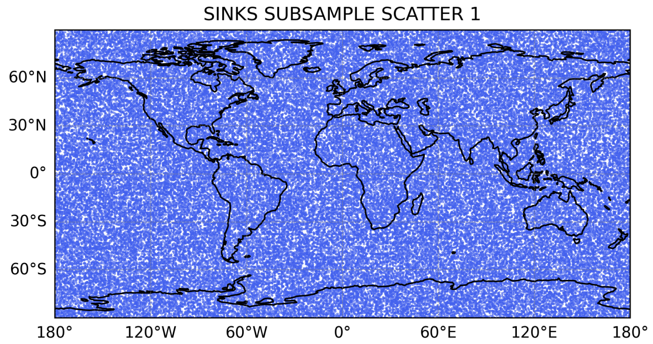

Supplementary Figure 6. Locations of the 100,000 randomly selected sinks of precipitation in the first and second subsample.

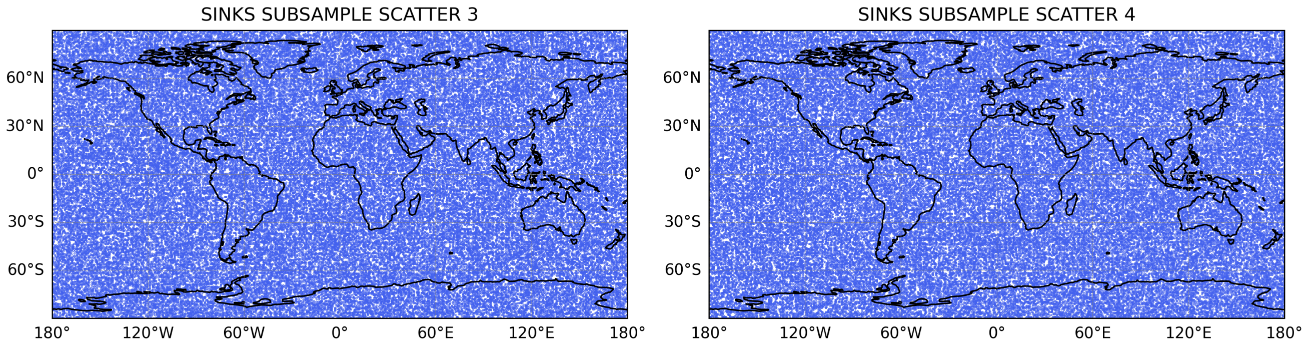

Supplementary Figure 7. Locations of the 100,000 randomly selected sinks of precipitation in the third and fourth subsample.

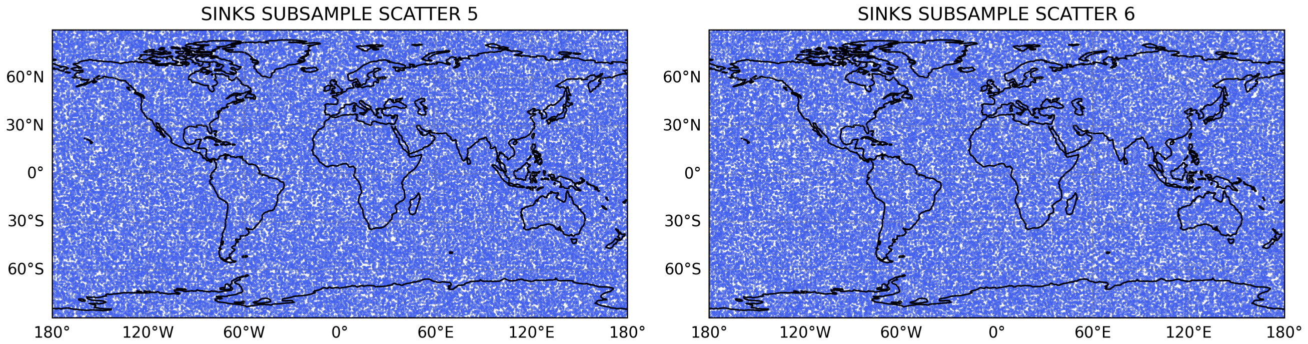

Supplementary Figure 8. Locations of the 100,000 randomly selected sinks of precipitation in the fifth and sixth subsample.

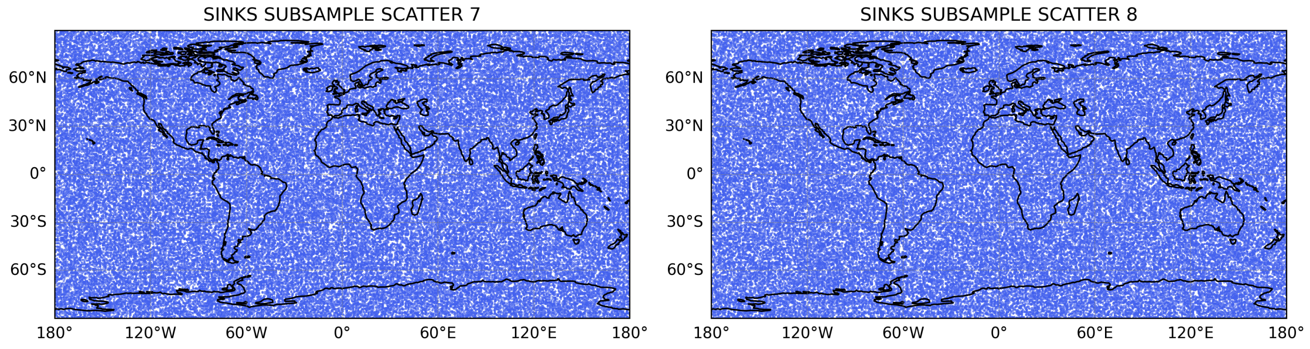

Supplementary Figure 9. Locations of the 100,000 randomly selected sinks of precipitation in the eighth and seventh subsample.

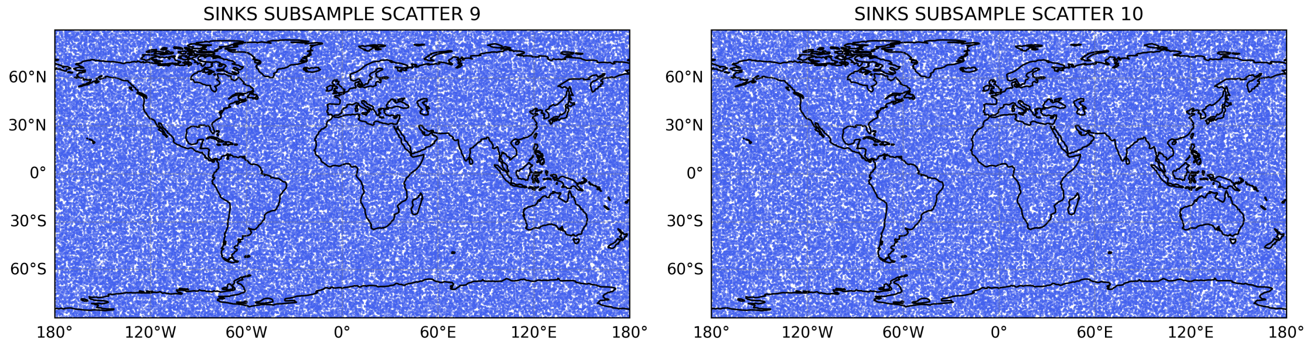

Supplementary Figure 10. Locations of the 100’000 randomly selected sinks of precipitation in the ninth and tenth subsample.
